# The Breakdown of the Hospital System

**Published:** 1889-03-02

**Authors:** 


### THE BREAKDOWN OF THE HOSPITAL SYSTEM.

UNDER this title, Dr. J. Francis Sutherland, the medical officer to H. M. prison at Glasgow, and formerly the resident physician at the British Hospital in Paris, has published a pamphlet which embodies the substance of a paper which he read before the British Medical Association at Glasgow, and another read at the opening meeting of the Glasgow Medico-Chirurgical Society. THE HOSPITAL cannot ignore a publication whose very title is an accusation against the institutions it advocates, and we publish a *résumé* of Dr. Sutherland's views as a prelude to collecting those of men whose interest in hospitals and knowledge of their management entitle them to speak on the subject. We invite correspondence, and shall gladly give publicity to all honestly-held opinions, without regard to any prejudices or preconceptions we may be supposed to hold. Free ventilation and open discussion never yet harmed a good cause, and very often those who think their ideas on any subject are most widely opposed, find when they have stated them explicitly that they are not only aiming at the same objects, ut moving towards them by roads that are parallel, or at any rate convergent. Hospitals are objects of real concern to three classes of the community—to the patients, actual and possible; to the subscribers, represented generally by the governors; and to the medical profession. All of these have that knowledge which gives them the right to speak; to all of them the columns of THE HOSPITAL are open.

Dr. Sutherland opens with the too-familiar fact that hospital incomes are very rarely equal to hospital expenditure. A system of finance which ends every year with a deficit cannot be looked upon as satisfactory; yet year by year hospital reports are published which show that the subscriptions and donations have not met the expenditure by a considerable amount, which in some cases reaches several thousands of pounds. As everyone knows, the London hospitals alone require an additional yearly income of £100,000 to enable them to do the work they are at present equipped for without running into debt; a similar depression, though perhaps less severe, characterises hospital finances all over the country. There are three ways by which the habitual deficit is met. Legacies inherited during the year are spent instead of being invested; the stock or investment fund is broken into; or money is borrowed from the bank. In the matter of legacies it sometimes happens that the whole of these is not utilised for current expenses, and so the capital account is increased; but as legacies come in varying and uncertain sums, even a superabundance of them, though it may set the yearly balance right, does not affect the cardinal fact that the reliable income of the hospital does not equal its average expenditure.

In the United Kingdom there are, in round numbers, 650 hospitals, containing 38,000 beds, or one bed for every 1,000 of the population. When, however, this total is divided, and the hospitals studied in regard to their location, it will be found that they do not exist in exact or even approximate proportion to the wants of the population. To take the extremes in towns, Dublin has one hospital bed to every 140 of its population; Leeds has not more than one to 1,020. In counties, the best provided in the kingdom is Midlothian, which has one bed to 410, while Durham on the other hand has but one to 1,930. Between these are all shades of proportion, that of London—with 90 hospitals, containing 10,000 beds, and a population of 4,200,000—being one in 420. Unless, therefore, we assume that the inhabitants of Dublin are three times as liable to diseases requiring hospital treatment as those of London, and more than eight times as much so as those of Leeds, it is evident that the proportion of hospital accommodation to population is unfair. Either Dublin has too much or Leeds has too little; and, indeed, both conditions may exist. Dr. Sutherland estimates that the total cost of the hospitals in the kingdom amounts approximately to £1,700,000. In making this calculation he estimates the cost per occupied bed at £45 a-year. This is certainly moderate, for if in some hospitals it is as low as £35, there are others where it amounts to £100. That such a wide variety of expenditure can exist in institutions similar in aim and constitution, implies extravagance in some, which would be investigated and put down if all were under the uniform control which could be given by a central administrative bureau. Returning to the question of hospital accommodation, it appears that in Scotland there are 17 counties, with an aggregate population of 725,000, without a single hospital bed. If schoo' and churches grew up in this haphazard fashion, what wculd be said? Would there not be an outcry that children were growing up in ignorance, adults living in heathenism, and an immediate demand for the state to step in and provide for the people what they could not provide for themselves? The care of the sick poor is as much a matter of national importance as these, yet it is left to the chances of benevolence, which result in rich districts having a sufficiency of hospital accommodation, or even more, while poor localities have the most inadequate provision, or none at all.

Of course it may be safely said that country districts do not require the same amount of hospital provision as towns, because density of population, disease, and death increase in direct proportion. But if it be laid down as a principle that hospital accommodation should bear a direct proportion to the death-rate of the district, because that is proportionate to the amount of sickness, it is still clear that hospital accommodation is unfairly distributed. In Glasgow the death-rate is 27 per thousand of the population, and the hospital accommodation is one to 590. In Edinburgh the death-rate is lower, and the hospital accommodation much more ample—one to 312. It is true that country districts send a considerable quota of patients to city hospitals, almost all severe surgical and obscure medical cases being sent for treatment, because presumably better attendance and more practised skill are to be found there. But this rural contingent is not so large as to justify the very considerable disproportion existing in different places between population, mortality, and hospital accommodation.

Further, it appears, says Dr. Sutherland, that even where the hospital accommodation is sufficient and even ample in proportion to the needs of the people, it is not always those who have the best right to hospital treatment who receive it. For this there are two reasons.

The rule in almost all hospitals is that, except in the case of accidents, applicants for treatment must bring a recommendation from a subscriber. Sometimes the number or price of these recommendations is fixed—six for a guinea, or something of the sort. Exceptions to the rule of the "subscriber's line" there are, such as the Birmingham hospitals and the Edinburgh Royal Infirmary; but a reference to hospital reports shows that in the large majority the recommendation is essential. Now, hospitals were founded primarily for the sick poor; those who now give or bequeath large sums to them mean their money to be expended on the sick poor. And it is by no means certain that those who can obtain the necessary "subscriber's line" are the most necessitous of the community. The probabilities are indeed the other way. The poorest of the sick are the least likely to command any influence over subscribers, and are often ignorant who these subscribers are. Others, comparatively better off, appeal to their employer, their clergyman, or someone else who can give or procure for them the necessary "line."

The second reason is that it has become the rule in most hospitals to select for admission to the wards only "interesting" cases—cases of uncommon diseases, and such as are distinctly curable. It is an all but invariable rule in hospitals to reject all applicants who are suffering from chronic or incurable disease. These cases would benefit by hospital treatment, the disease would be arrested, and the patients' suffering relieved; but the rules forbid their being received, and the doctor who examines patients in the waiting-room is expected to select from the candidates for admission only those who are likely to be cured or materially benefited by hospital treatment. The sick poor do not invariably suffer from "interesting" diseases, they are very liable to chronic and incurable complaints; therefore, a very large proportion of them are ineligible for the voluntary hospitals. The only exceptions to this rule are the fortunate patients who come armed with a line from a well-known subscriber.

What are the sick, who are refused admittance to the hospitals for either of these reasons, to do? They can either remain in their homes, under conditions that retard or prevent their recovery, and lacking such food and comforts as are absolute necessaries in their condition, or they can go into the workhouse infirmaries, which are often inferior to the hospitals both in nursing and sanitary conditions, and which, moreover, place on all who enter them the stigma of pauperism. Practically, the people who enter the workhouse infirmaries are of the same class as those who are received into the voluntary hospitals; and a difference in the character of their complaints should not be accompanied by such a difference in their treatment by the community. "I think the time has come," says Dr. Sutherland, "when the parochial hospital should be divorced from the poorhouse, and be as much a part of our hospital system as our infirmaries, etc."

Meanwhile, though a large number of cases which would be benefited by hospital treatment are rejected for the reasons above given, the wards are full, and hospital managers are forced to turn away others which they regard as suitable because they have not a bed to give them. Clearly then, except in a few localities, the hospital accommodation is insufficient to the needs of the population. Another indication that this is so may be taken from the mortality tables. There is more sickness among the poor than among the rich, and certainly more deaths, yet in our large cities only about 5 per cent. of the deaths take place in the hospitals. As some, at least, of these may be assumed to be the serious cases sent from the country, only about 4 per cent. of the town deaths ought to be credited to the hospitals. From 5 to 11 per cent. occur in the workhouse infirmaries. In Paris about 20 per cent. of the deaths occur in the hospitals.

In connexion with this, it is interesting to learn how many deaths occur in their own homes among the class from whom hospital patients are drawn. From a classification of the houses in Glasgow at the last census, Dr. Sutherland finds that 70 per cent. of the population live in dwellings of one or two rooms, such as are rented at £10 a-year or less in our large towns. This may be regarded as the hospital patient class. And, according to the calculations of Dr. Littlejohn (the medical officer of health for Edinburgh), 52 per cent. of deaths occur in houses rented under £10. Doubtless hospital treatment cures many who would have died at home, but if this be so it is the more to be regretted that the accommodation, which might mean the saving of life, is not available for the sick poor.

The evidence collected by Dr. Sutherland, supported as it is by carefully-compiled statistics, has led him to believe: First, that the amount of hospital accommodation at present provided is inadequate to the needs of the population; and, secondly, that such accommodation as is provided is not utilised strictly and solely by those whose circumstances justify their reception as hospital patients. Also, that this latter fact is caused in part by the fact that patients recommended by subscribers have a preference over others, though these do not always belong to the class from whom hospital patients should be drawn—the necessitous, but, while in health, self-supporting poor.

For these defects he proposes the following remedies:—

That the system of subscribers' lines should be given up, and admission to the hospital based entirely on suitability of disease and circumstance.

That the workhouse infirmaries should be united to the hospitals, but regarded as specially adapted for chronic and incurable cases.

That all should be under the control of a central administrative bureau, composed of delegates from the subscribers, from corporate bodies, urban and rural, from the working classes, and from the Crown (the last to be medical men), presided over by a Minister of Health; and that the foundation of small special hospitals should be discouraged, as the work undertaken by them could be as well performed in the special wards of a large hospital, while that part of the subscriptions of the public which is now expended in their management would be made available for the relief of the sick poor. The central authority would establish hospitals where the provision was inadequate, and would be able to bring about a greater uniformity, and consequent economy in hospital expenditure.

That as philanthropy, though now taxed to the uttermost, cannot provide sufficient hospital accommodation, the State ought to take up the matter and make up the necessary amount, the care of the sick poor being a national duty. That this should be done by subventions from the Treasury, which are not to take the place of private or local benevolence, but to supplement it; the grants in aid of hospitals in each district being strictly proportionate to the money contributed by the district.

That the central authority should establish a medical bureau, where the examination of applicants for hospital treatment should be conducted by practitioners of experience, while an inquiry would be made concerning their circumstances.

That convalescent homes and sanitoria should be established in the country and at the seaside in larger numbers than at present. The existence of these would often enable patients to be dismissed from the hospital sooner than at present. In many cases a patient remains in the hospital when his disease is practically cured, because he has not yet regained sufficient strength to enable him to resist his frequently unfavourable home surroundings.

That pay wards should be attached to general hospitals.

The recommendation of assistance by Parliamentary grants, however limited and conditional, is sure to provoke comment. But fortunately we have the means of investigating the system on its practical merits. Not only is it carried out in France, the State of New York, and in our Australian colonies; but in Dublin there are nine hospitals maintained in the dual manner Dr. Sutherland recommends, by State grants supplementing voluntary subscriptions.

We have stated Dr. Sutherland's opinions and recommendations clearly and briefly. Let those who have studied the question of hospital work and hospital needs say what they think of them.

